# Age and gender differences in ACE2 and TMPRSS2 expressions in oral epithelial cells

**DOI:** 10.1186/s12967-021-03037-4

**Published:** 2021-08-19

**Authors:** Jinfeng Peng, Jiwei Sun, Jiajia Zhao, Xuliang Deng, Fengyuan Guo, Lili Chen

**Affiliations:** 1 Department of Stomatology, Union Hospital, Tongji Medical College, Huazhong University of Science and Technology, Wuhan, 430022 China; 2 School of Stomatology, Tongji Medical College, HuazhongUniversity of Science and Technology, Wuhan, 430030 China; 3 Hubei Province Key Laboratory of Oral and Maxillofacial Development and Regeneration, Wuhan, 430022 China; 4 Department of Geriatric Dentistry, Peking University School and Hospital of Stomatology, Beijing, 100081 China

**Keywords:** Coronavirus, Gene expression, Gender differences, SARS-CoV-2, Bioinformatics

## Abstract

**Background:**

SARS-CoV-2, which has brought a huge negative impact on the world since the end of 2019, is reported to invade cells using the spike (S) protein to bind to angiotensin-converting enzyme II (ACE2) receptors on human cells while the transmembrane protease serine 2 (TMPRSS2) is the key protease that activates the S protein, which greatly facilitates the entry of SARS-CoV-2 into target cells. In our previous study, it was observed that the positive rate of SARS-CoV-2 nucleic acids in saliva was higher in male and the elderly COVID-19 patients, suggesting that the susceptibility of oral tissues to SARS-CoV-2 may be related to gender and age. This research aimed to further investigate the SARS-CoV-2 susceptibility in oral tissues and influencing factors from the perspective of ACE2 and TMPRSS2, which were two proteins closely associated with SARS-CoV-2 infection.

**Methods:**

Immunofluorescence was used to find the localization of ACE2 and TMPRSS2 in oral mucosal tissues. Transcriptomic sequencing data of several datasets were then collected to analysis the relationship between the expressions of ACE2 and TMPRSS2 with the age and gender of patients. Furthermore, oral tissues from patients with different ages and genders were collected. Immunohistochemistry staining, qRT-PCR and western blot were performed to explore the relationship between expression levels of ACE2 and TMPRSS2 and patient age as well as gender.

**Results:**

The results showed that the two proteins were able to be co-expressed in the epithelial cells of oral tissues, and their expression levels were higher in the relatively elderly group than those in relatively younger group. Male oral epithelial cells exhibited higher level of TMPRSS2.

**Conclusions:**

Our findings comprehensively confirmed the existence of ACE2 and TMPRSS2 in oral tissues and clarify the relationship between the expression levels with human age and gender for the first time, providing evidence for possible entry routes of SARS-CoV-2 and the influencing factors of SARS-CoV-2 colonization in oral cavity. Thus, the oral mucosa might be at potential risk of infection by SARS-CoV-2, especially in male or elderly patients. Using saliva to detect the nucleic acids of SARS-CoV-2 may be more accurate for elder male COVID-19 patients.

**Supplementary Information:**

The online version contains supplementary material available at 10.1186/s12967-021-03037-4.

## Background

From the end of 2019 till now, the emergence of SARS-CoV-2, which can cause a potentially life-threatening viral respiratory disease named COVID-19, has brought a tremendous impact on people around the world. By 23 March 2021, the cumulative number of cases worldwide has reached 122.5 million [1]. The mainstream view on the process of SARS-CoV-2 invasion in the human body is that the spike (S) protein plays a key role in the receptor recognition and cell membrane fusion, which consists of S1 and S2 subunits. The S1 subunit contains a receptor-binding domain that recognizes and binds to the host cells, while the S2 subunit mediates viral cell membrane fusion [2, 3]. The angiotensin converting enzyme 2 (ACE2) is the most widely recognized portal target of SARS-CoV-2 invasion [4–6]. Subsequent studies confirmed that the transmembrane protease serine 2 (TMPRSS2) is used for S protein activation, thus strengthening the binding of SARS-CoV-2 and ACE2, and the invasion of SARS-CoV-2 can be blocked by TMPRSS2 inhibitor [7–9]. These studies suggest that cells expressing ACE2 and TMPRSS2 may be more susceptible to SARS-CoV-2 infection.

Oral cavity is an important route for pathogens to invade the human body. It has been reported that the ACE2 and TMPRSS2 can be expressed in oral tissues such as tongue epithelium, salivary glands, tongue, gingival tissues and human fungiform papillae taste cells [10–12]. Moreover, we have found that SARS-CoV-2 could be detected in saliva by collecting saliva from COVID-19 patients and dry mouth as well as amblygeustia could be considered as initial symptoms of SARS-CoV-2 infection [12], which means oral tissues are most likely an important entry route for SARS-CoV-2, and the susceptibility of oral epithelial cells to SARS-CoV-2 may also be related to the expression levels of ACE2 and TMPRSS2. At present, the detection potential of saliva and the oral symptoms of COVID-19 patients have attracted increasing attention and extensive research. However, few articles have focused on the differences in SARS-CoV-2 susceptibility of oral epithelial cells in different human groups.

Here, we aim to figure out the influencing factors of the SARS-CoV-2 susceptibility in oral tissues by analyzing the expression levels of ACE2 and TMPRSS2 in people with different ages and genders. Firstly, we have clarified the ACE2 and TMPRSS2 mainly exist in oral epithelial cells of oral mucosal tissues. Transcriptomic sequencing data of several datasets from TCGA and GEO were then collected to figure out that the expression levels of ACE2 and TMPRSS2 are concerned with the age and gender of patients. Furthermore, we have collected oral tissues from patients with different ages and genders to explore the relationship between expression levels of ACE2 and TMPRSS2 and human age as well as gender. The results reveal that the expression levels were higher in relatively elderly people than in relatively younger people. Meanwhile, male oral epithelial cells exhibit higher level of TMPRSS2 than female with similar age. Hence, the oral mucosa might be at potential risk of infection by SARS-CoV-2, providing theoretical evidence for the occurrence of oral symptoms in COVID-19 patients, especially in male or elderly patients, which may bring a new insight into future explorations of the SARS-CoV-2 susceptibility in oral epithelial cells and its mechanisms.

## Materials and methods

### Specimen collection

A total of 49 normal oral mucosal tissues, including the tissues of the tongue, the mouth floor, the gingiva, the buccal mucosa and the upper palate, originated from operations of stomatology surgery, were collected between Jun 2019 and Oct 2020. All patients signed an appropriate consent form for biobanking. There were 33 males and 16 females, ranging from 27 to 77 years old. In order to make the sample size of different groups as sufficient as possible and thus make the experimental data more representative, we defined the population over 50 years old as the relatively elderly group according to the age of samples collected. General information of the clinical samples is shown in Additional file 2.

### Acquisition of ACE2 and TMPRSS2 relative expression

Bioinformatic analysis was carried out to figure out relative expression level of ACE2 and TMPRSS2. Transcriptomic profiling data from GSE9844, GSE30784, GSE42743 were downloaded by R package GEOquery. Transcriptomic sequencing data of adjacent normal tissues from OSCC patients in TCGA were downloaded with the help of TCGAbiolink. Relative expression of ACE2 and TMPRSS2 were selected out for further exploration. General information of the clinical samples is shown in Additional file 2.

### Description of immune infiltration landscape

The method ssGSEA was selected to quantify the relative number of 28 types of immune cells based on expression status of immune-related genes. R package pheatmap was applied for visualization of immune infiltration status among different samples in TCGA dataset.

### Gene ontology enrichment exploration

The median expression level of ACE2 and TMPRSS2 in samples from TCGA dataset were regarded as criteria for division of high or low expression subgroups for further enrichment analysis. R package clusterProfiler was used for gene ontology enrichment analysis in comparison of above high and low expression subgroups, based on their transcriptomic data from TCGA dataset.

### Real-time quantitative polymerase chain reaction (qRT-PCR) analysis

Total RNA from tissues was isolated using RNA isolater Total RNA Extraction Reagent (R401-01, Vazyme, China) according to the manual instruction and reversed to cDNA using HiScript III RT SuperMix for qPCR (Vazyme). A 1-μl volume of cDNA was amplified in a 10-μl volume reaction system with the ChamQ SYBR qPCR Master Mix (Vazyme). qRT-PCR was complemented using SYBR Green PCR protocol on a real time PCR system (ABI 7300, Applied Biosystems, USA). Relative mRNA expression levels of the target genes were normalized against the mRNA expression level of *GAPDH* and calculated via the 2^−△△CT^ method. The primers used for amplification are listed in Additional file 1.

### Western blot analysis

Protein samples were obtained from tissues utilizing RIPA lysate (P0013B, Beyotime, Shanghai, China) and ultrasonic oscillation. After denaturation by sodium dodecyl sulfatepolyacrylamide gel electrophoresis (SDS-PAGE) loading buffer, proteins were fractionated via SDS-PAGE and transferred onto the 0.45-μm polyvinylidene fluoride (PVDF) membranes. The membranes were then blocked with 5% bovine serum albumin (BSA, A1933, Sigma-Aldrich) for 1 h at room temperature and incubated overnight with primary antibodies against ACE2 (Proteintech, 21115–1-AP, 1:1000), TMPRSS2 (Abclonal, A9126, 1:1000) or GAPDH (Proteintech, 10494–1-AP, 1:10000) at 4 °C. On the following day, the membranes were washed three times with TBST before reprobed with a secondary goat anti-rabbit antibody (Proteintech, SA00001-2, 1:2000) for 1 h at room temperature. Next, the membranes were exposed to autoradiography film (Amersham, Little Chalfont, UK) using Immobilon Western Chemiluminescent HRP Substrate Reagent (WBKLS0500, Millipore, USA). Image J software was used to quantify the gray values of immune response bands. Relative protein expression levels of the target proteins were normalized against the expression level of GAPDH.

### Immunohistochemistry and immunofluorescence

After PFA for 24 h, decalcified in 10% ethylenediaminetetraacetic acid for 4 weeks, and embedded in paraffin, Sects. (5-μm-thick) were stained with immunohistochemical and immunofluorescence staining. For immunohistochemical staining, the sections were pre-treated with sodium citrate buffer (pH6, epitope retrieval solution 1) for 20 min for antigen retrieval and then incubated with anti-ACE2 antibody (Abclonal, A12737, 1:100) and anti-TMPRSS2 antibody (Abclonal, A9126, 1:100) for 15 min at room temperature, and subsequently detected with a horseradish peroxidase (HRP)-conjugated compact polymer system (Dako/Agilent, Santa Clara, CA, USA). For immunofluorescent staining, the sections were pre-treated with graded ethanol (100%、95%、75%) for 5 min each. After repaired with high temperature and high pressure, the sections were washed with TBS and blocked with 5% bovine serum albumin. Anti-ACE2 antibody (Abclonal, A12737, 1:200) was applied to the slides and incubated at 4 °C overnight. After three washes with TBS, anti-TMPRSS2 antibody (Abclonal, A9126, 1:200) was applied to the slides and incubated at 4 °C overnight. After three washes with TBS, the slides were incubated with Alexa-Fluor 488-conjugated goat anti-rabbit and Alexa-Fluor cy3-conjugated goat anti-rabbit secondary antibody. Nuclei were stained with DAPI. Images were taken using a confocal laser scanning microscope.

### Statistical analysis

Statistical analyses were performed with GraphPad Prism software version 8.0. All data were presented as mean ± standard deviation (SD) values. The two-tailed Student’s t test was used to evaluate the differences in the expression levels of ACE2 and TMPRSS2 in different age and gender groups. Data were inferred as statistically significant if *P* values were < 0.05.

## Results

### Co-expression of ACE2 and TMPRSS2 in oral epithelial cells

**Fig. 1:**
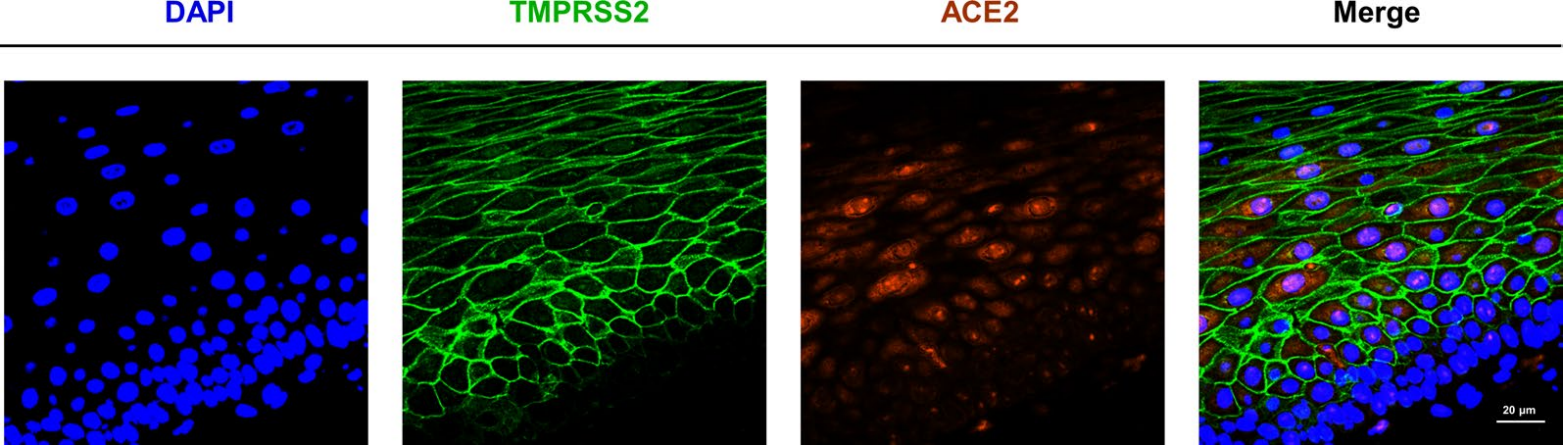
Co-expression of ACE2 and TMPRSS2 in oral epithelial cells. Immunofluorescent images of epithelial cells in oral tissues stained with DAPI (blue), TMPRSS2 (green) and ACE2 (red). Scale bar, 20 μm

As oral epithelium acts as a barrier against external substance, presence of SARS-CoV-2 in saliva samples from diagnosed patients firstly led us to consider the susceptibility of oral epithelial cells to the virus. Immunofluorescent images showed that both of the two most important molecular adaptors associated with infection of SARS-CoV-2, ACE2 and TMPRSS2, were expressed in the oral epithelial cells. And the ACE2 expressed mainly intracellular, including cytoplasm and nucleus, while TMPRSS2 was mainly localized in the cell membranes of oral epithelial cells (Fig. 1). This result confirmed the hypothesis that oral epithelium might be exposed to the risk of SARS-CoV-2 infection because of the co-expression of these two key proteins that mediate viral invasion of cells.

### Bioinformatic analysis of ACE2 and TMPRSS2 in different cohorts

**Fig. 2:**
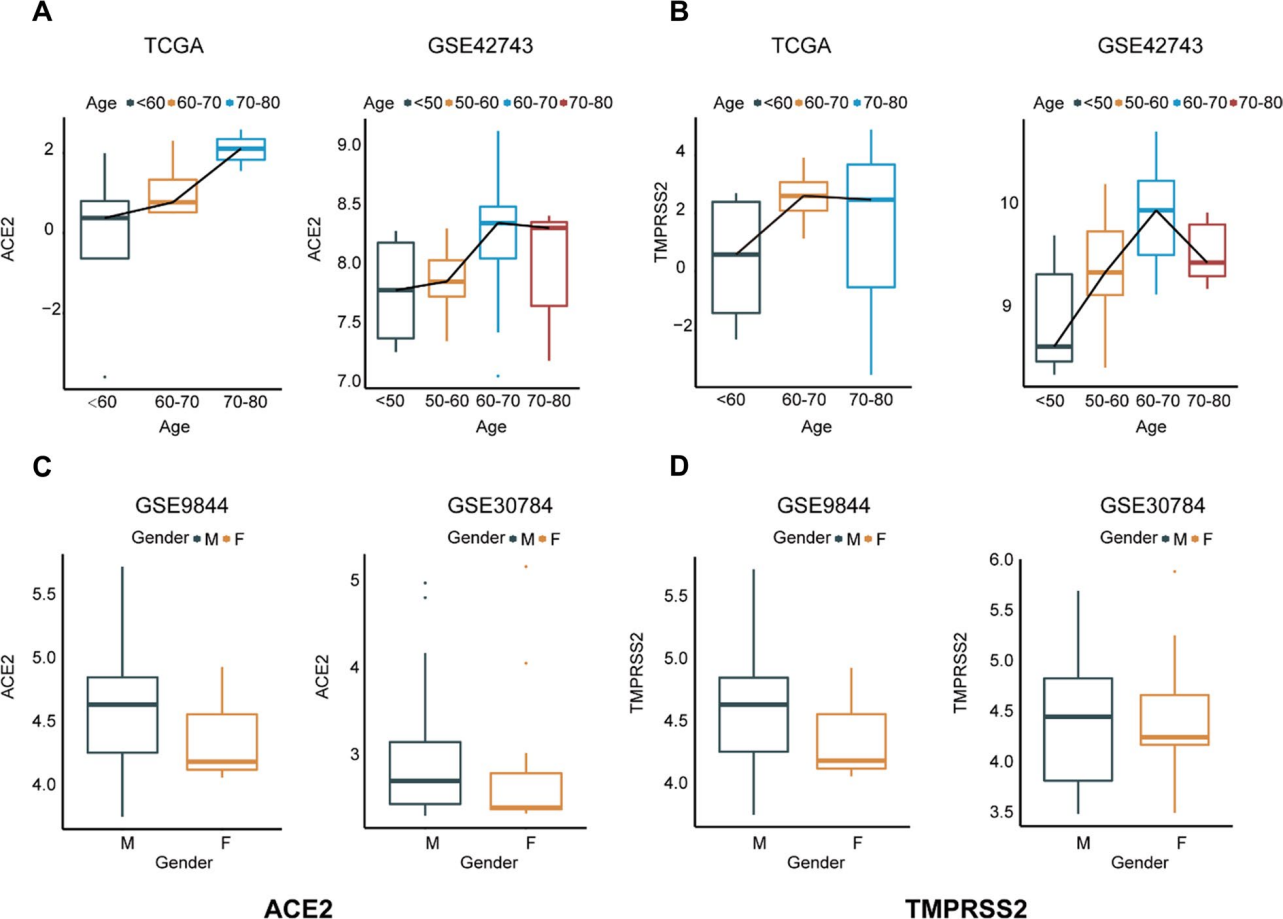
Bioinformatic analysis of ACE2 and TMPRSS2 in different cohorts.** A**,** B** Relative expression levels of ACE2 and TMPRSS2 between oral epithelial samples among cohorts at different ages from datasets TCGA and GSE42743. ** C**, ** D** Relative expression levels of ACE2 and TMPRSS2 between oral epithelial samples of male and female cohorts from datasets GSE9844 and GSE30784

In one of our previously published studies, we confirmed the presence of SARS-CoV-2 by collecting saliva from COVID-19 patients. Based on the clinical information from 17 patients confirmed with COVID-19, it was observed that 4 of them showed positive response of saliva SARS-CoV-2 detection. Positive rate of saliva SARS-CoV-2 from male patients reached at 66.7%, higher than that from female patients, which was just 20%, and the average age of saliva SARS-CoV-2 positive patients was at 71, higher than 63, the average age of all patients [12]. This phenomenon led us to explore its possible explanations. As ACE2 and TMPRSS2 were reported as significant adaptors for invasion of SARS-CoV-2 virus into human epithelium, expression of the two molecules in human oral epithelium were then explored using bioinformatic analysis. In perspective of age, samples in TCGA and GSE42743 datasets showed an increasing expression of both ACE2 and TMPRSS2 (Fig. 2A, B). Meanwhile, ACE2 and TMPRSS2 expression level indicated an amounting tendency in male oral epithelium compared with that from female in GSE9844 and GSE30784 (Fig. 2C, D). Higher expression levels of ACE2 and TMPRSS2 in male and older cohorts just coincided with higher saliva positive rate of SARS-CoV-2.

### Variation of cell functions and immune infiltration along with ACE2 and TMPRSS2 expression

**Fig. 3:**
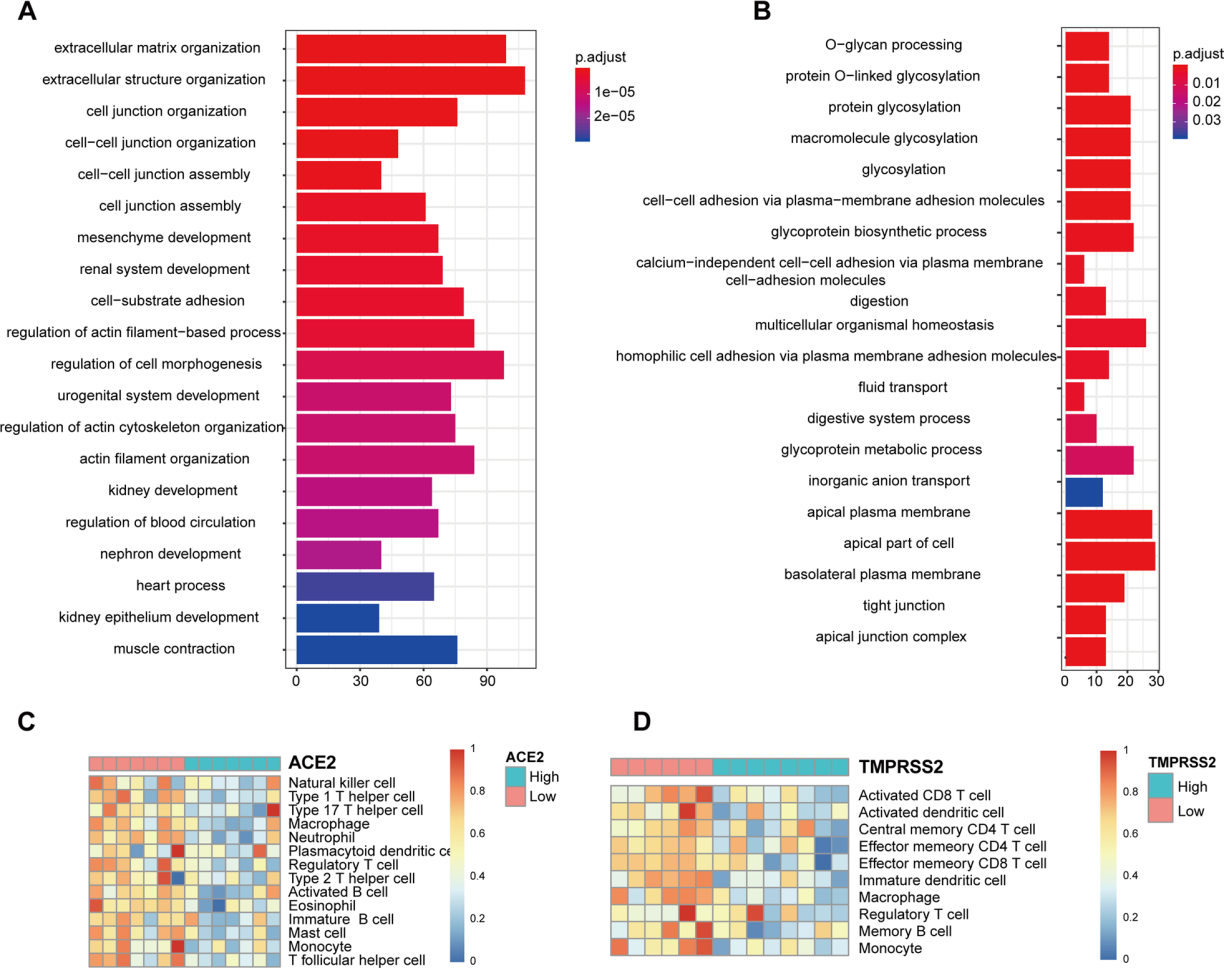
Variation of cell functions and immune infiltration along with ACE2 and TMPRSS2 expression. ** A**, ** B** Gene ontology (GO) enrichment analysis of transcriptomic sequencing data of filtered oral epithelial samples from TCGA dataset. According to the median expression level of ACE2 and TMPRSS2, samples from TCGA dataset were divided into two subgroups for further comparison. ** C**, ** D** Description of immune infiltration status of oral epithelium samples from TCGA dataset. Expression levels of ACE2 and TMPRSS2 were used as standard for division of subgroups

To explore difference of cell function and behavior as well as immune infiltration between oral epithelium with higher and lower expression levels of ACE2 and TMPRSS2, gene ontology (GO) enrichment analysis was first carried out. ACE2 expression level was associated with multiple GO terms linked to epidermal variations, including epidermal cell differentiation, epidermis development, keratinocyte differentiation and cornification (Fig. 3A). In terms of TMPRSS2, it was related to cell adhesion, such as cell–cell adhesion via plasma-membrane adhesion molecules, homophilic adhesion via plasma membrane adhesion molecules, tight junction as well as typical junction complex (Fig. 3B). When it comes to immune infiltration status, the infiltration of congenital immune cells, including neutrophils, macrophages, monocytes and dendritic cells, varied along with the expression of ACE2 and TMPRSS2. As for acquired immune cells, T helper cell type 1, T helper cell type 2 and T helper cell type 17, regulatory T cells, follicular helper T cells, memory T cells, natural killer cells and memory B cells also showed functional differences with the expression variations of ACE2 and TMPRSS2. When the relative expression of ACE2 and TMPRSS2 increased, the infiltrating degree of all these cells tended to decrease, suggesting that the expression of these two proteins was related to the congenital and acquired immunodeficiency. According to the gene ontology (GO) enrichment analysis in Fig. 3, the immune infiltration status of oral epithelial specimens in the TCGA dataset may give a clue that the higher levels of ACE2 and TMPRSS2 in oral epithelium, the poorer the adaptive antiviral immune response was. (Fig. 3C, D).

### Multiple biological tests confirm that the expression levels of TMPRSS2 and ACE2 in oral mucosa are different by age or gender

**Fig. 4:**
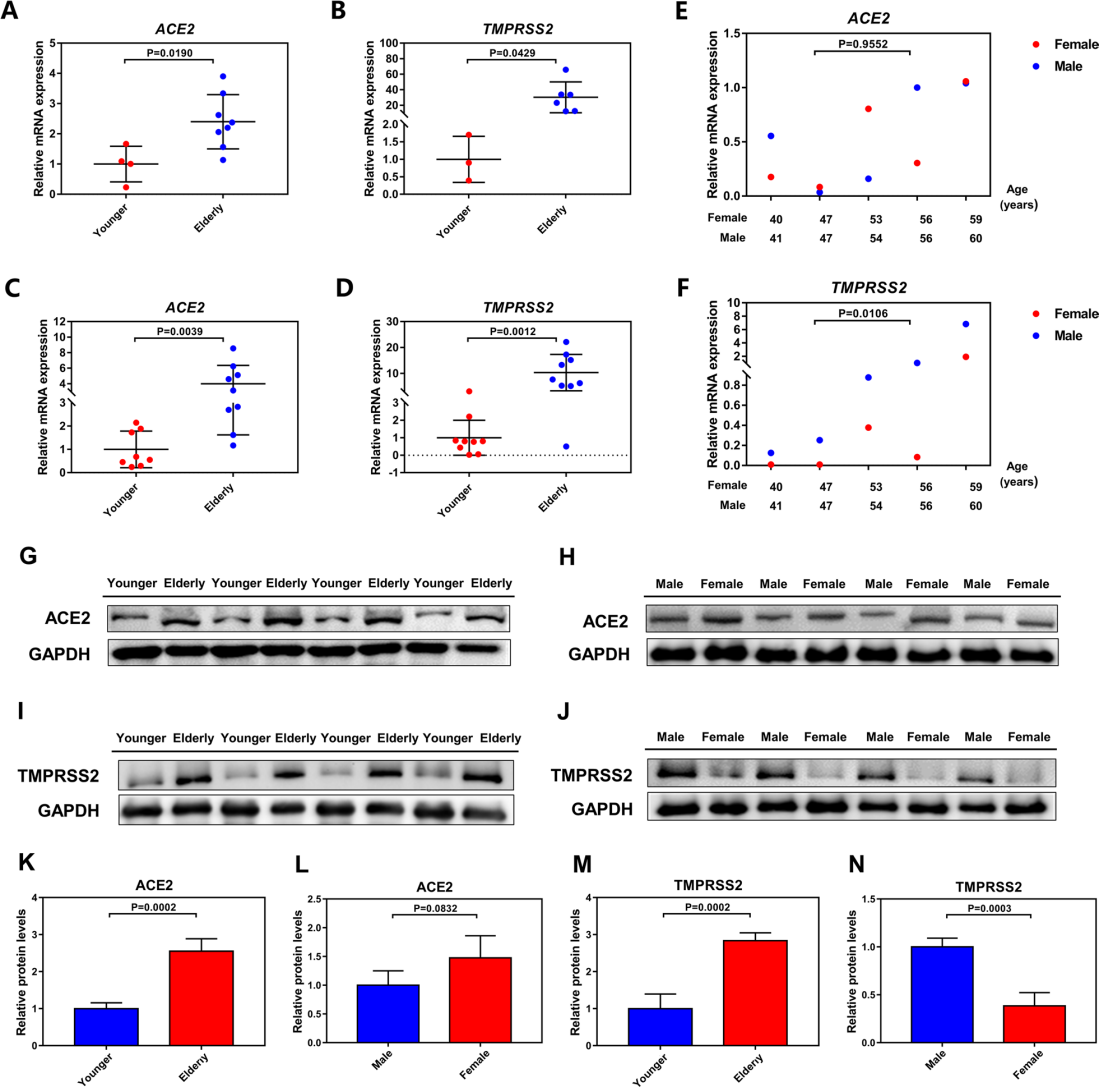
Quantitative analysis showed that the expression levels of ACE2 and TMPRSS2 in oral mucosa tissues were different by age or gender.** A**, ** B** Real-time quantitative polymerase chain reaction (qRT-PCR) analysis showed the relative mRNA levels of *ACE2* and *TMPRSS2* in oral mucosal tissues of the relatively younger and relatively elderly females. ** C**, **D** qRT-PCR analysis showed the relative mRNA levels of *ACE2* and *TMPRSS2* in oral mucosal tissues of the relatively younger and relatively elderly males. **E**, **F** qRT-PCR analysis showed the relative mRNA levels of *ACE2* and *TMPRSS2* in oral mucosal tissues of females and males with similar age. **(G-H)** Western blot analysis showed the protein levels of ACE2 in people of different ages and genders. **I**–**J** Western blot analysis showed the protein levels of TMPRSS2 in people of different ages and genders. **K**–**N** showed the statistical analysis of **G**–**J** A–N, n ≥ 3. Data were analyzed using two-tailed Student’s t test and presented as the mean ± SD. *P* values were provided in case of multiple samples, and *P* values < 0.05 indicated statistical significance

**Fig. 5:**
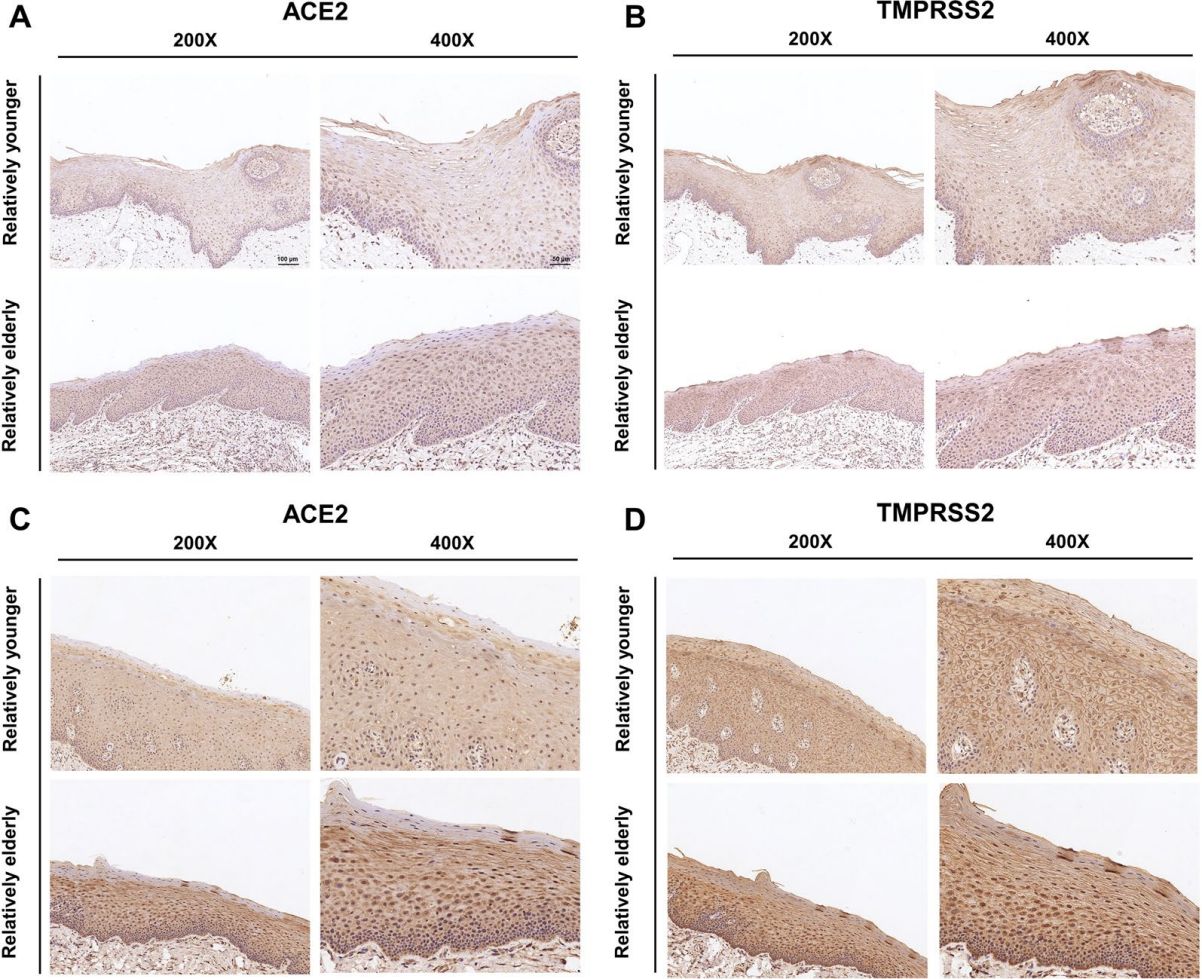
Qualitative analysis showed that the expression levels of TMPRSS2 and ACE2 in oral mucosa were different by age or gender. **A**, **B** Immunohistochemistry of ACE2 and TMPRSS2 in oral mucosal tissues of the relatively younger and relatively elderly females. **C**, **D** Immunohistochemistry of ACE2 and TMPRSS2 in oral mucosal tissues of the relatively younger and relatively elderly males. **A**–**D**, n = 3. Scale bar, ×200, 100 μm, ×400, 50 μm

Based on the above results of co-localization and bioinformatics analysis, to further verify the expression levels of ACE2 and TMPRSS2 in human oral mucosal tissues and their relationship with age and genders, we have collected human oral mucosal tissue samples with different ages and genders, and conducted a variety of biological tests. qRT-PCR was used to measure the relative mRNA levels, and it was found that the expression levels of *ACE2* and *TMPRSS2* in oral mucosa were significantly increased in relatively elderly women than in relatively younger women (Fig. 4A, B), which was also found in oral mucosal specimens from the males of different ages (Fig. 4C, D). To determine the correlation between the expression levels and the gender, we examined the relative mRNA levels of *ACE2* and *TM*PRSS2 in oral mucosal tissues of females and males with similar age. Our findings showed that there was no difference in the mRNA expression of *ACE2* in oral tissues between males and females of similar age (Fig. 4E). However, the mRNA expression of *TMPRSS2* in female oral tissues was lower than that of males with similar age (Fig. 4F). To more accurately evaluate the expressions of ACE2 and TMPRSS2 in people of different ages and genders, we measured the protein expression levels through western blot and found that, in general, the protein levels of ACE2 in oral mucosa of the elderly were higher than that of the younger (Fig. 4G, K), but there was no significant difference between the females and males with similar age (Fig. 4H, L). And the protein levels of TMPRSS2 were also found to increase in oral mucosal tissues of both the relatively elderly and the males compared with the younger and the females of similar age (Fig. 4I, J, M, N). The expressions of ACE2 and TMPRSS2 in the oral mucosal tissues were further assessed by immunohistochemistry, which revealed that in both females and males, ACE2 and TMPRSS2 were more expressed in the relatively elderly than the younger (Fig. 5A–D). The above results suggest that ACE2 and TMPRSS2 are indeed expressed in human oral mucosal tissues, and their expression levels are higher in the relatively elderly group than in relatively younger group. Meanwhile, the expression level of TMPRSS2 in oral mucosal tissues of males is higher than that of females with similar age.

## Discussion

Colonization and invasion of pulmonary tissues was the most significant characteristic for SARS-CoV-2 [13]. However, recent studies have confirmed SARS-CoV-2 can be detected in alveolar lavage fluid, nasal secretions, sputum, urine, feces, and blood samples of COVID-19 patients [14, 15], which could just explain the respiratory, gastrointestinal and reproductive system symptoms observed in COVID-19 patients [16–19]. Through single-cell sequencing and RNA-seq, researchers found that ACE2 was mainly expressed in the lung, kidney, testis and intestinal tissues [20–24], while TMPRSS2 was able to be co-expressed with ACE2 in nasal epithelial cells, lung and bronchial branches [25]. This may explain the tissue tendency of SARS-CoV-2 to some extent. In previous studies, we have found that dry mouth and amblygeustia were two major oral symptoms of COVID-19 patients [12]. By immunofluorescence co-localization, we confirmed the presence and localization of ACE2 and TMPRSS2 in oral tissues, and they were mainly expressed in the superficial oral epithelial cells, rather than in the deep submucosal tissues. This result provided possibility that SARS-CoV-2 might colonize and invade oral epithelium via S protein mediated by ACE2 and TMPRSS2, resulting in the damage to salivary glands and taste buds. Therefore, a clearer understanding of the expression status of ACE2 and TMPRSS2 would be extremely helpful in exploring the susceptibility of oral epithelial cells to SARS-CoV-2.

In this study, through GO enrichment analysis, ACE2 was found to be associated with epidermal variations and TMPRSS2 was related to cell adhesion. Besides, higher levels of ACE2 and TMPRSS2 might indicate poorer adaptive anti-virus immune responses. Those results indicated that ACE2 and TMPRSS2 expression might pose influence on cell differentiation and adhesion of oral epithelium, which might further contribute to susceptibility of SARS-CoV-2. The overexpression of ACE2 and TMPRSS2 in oral epithelium might decrease its anti-virus immune potential, further enhancing the risk of oral SARS-CoV-2 colonization.

Bioinformatics analysis of ACE2 and TMPRSS2 in different cohorts and a variety of biological tests of oral mucosal tissues revealed that male or relatively elderly patients seem to show higher levels of ACE2 and TMPRSS2 expression. From clinical perspective, oral epithelium of male or elderly cohorts might be much easier to get infected by SARS-CoV-2 through oral-fecal transmission. Thus, protection measures for such populations should be maintained at a high level to avoid possible transmission of the virus. However, in the statistical analysis of the data from the database, there was no statistically significant difference in the expression levels of ACE2 and TMPRSS2 in oral epithelial tissues between different gender groups (*P* > 0.05). This may be due to the insufficient number of samples in the databases. When selected samples were divided into different groups, the number of samples in each group is even less, making the difference between each group unobvious. Therefore, it is difficult to get the conclusion of statistically significant difference.

To effectively restrain the spread of SARS-CoV-2, fast and accurate detection strategies are still in great need. Up till now, RT-PCR of virus RNA has still been the most efficient and prevalent way for detection of SARS-CoV-2 compared with rapid antigen detection (RAD) tests and other immunoassays [26, 27]. Clinical samples from nasopharyngeal or oropharyngeal swabs were widely selected as detection tools for them [28, 29]. However, this approach is not applicable in some cases, such as poor nasal anatomy or patients who cannot tolerate it [30]. In the meanwhile, that process often makes people cough or gag, increasing the risk of health care workers being infected with the virus. Therefore, clinical experiments were carried out to figure out more readily available samples for detection. Current studies revealed that various types of biological samples from COVID-19 patients, including blood, urine, saliva as well as fecal and anal swabs exhibited detectable levels of SARS-CoV-2 nucleic acids [12, 31, 32]. Among them, as one of the most readily accessible and easily collected bodily fluids, saliva has become a studying focus with great potential. Lots of studies have focused on efficiency of saliva detection, but the sensitivity of salivary viral RNA ranged from 66 to 92% among different studies [33]. Figuring out reasons for this diversity was necessary, and clinical background was assumed to be the most important part [34]. In our study, we found the expression levels of ACE2 and TMPRSS2 in the oral epithelial cells of the relatively elderly people were higher than those of the relatively younger people, and the expression of TMPRSS2 in the oral epithelial cells of males was higher than that of females with similar age, which may explain the higher positive rate of SARS-CoV-2 nucleic acids in male and the elderly COVID-19 patients in the saliva test report we published earlier [12]. This phenomenon suggested that clinical background, especially gender and age, might influence salivary viral load and subsequent detection accuracy for COVID-19 patients. Saliva detections for COVID-19 patients seem to be more accurate for male and elderly cohorts, whose oral epithelial cells may be more susceptible to SARS-CoV-2 invasion. Furthermore, it needs to be emphasized that more attention should be paid to infected patients who are male or at higher age since they may have greater risk of spreading SARS-CoV-2 via saliva.

Specifically,, the expression of ACE2 did not show gender difference in the following biological tests. This may be due to the fact that the sample size we collected is not enough. With the increase of the sample size, the individual's accidental error decreases, which may lead to the statistical significance of the overall difference [35]. At the same time, there are also individual differences and tissue differences in gene expression [36, 37]. The samples include a variety of oral tissues, including tongue, the mouth floor, the gingiva, the buccal mucosa and the upper palate. The expression differences among different tissues may also be one of the reasons for the non-statistical difference in the results. At present, most of the oral tissue specimens are collected from maxillofacial surgery. Due to the obvious gender difference in morbidity, female specimens are generally less than male specimens.

It should be emphasized that the predominantly descriptive (due to small sample size) and hypothesis-generating role are two major limitations of the study. In this study, we mainly made a statistical analysis of the public database and completed further clinical sample verification, and found that expression levels of two molecules (ACE2 and TMPRSS2), which are currently reported to be related to the SARS-CoV-2 infection, are associated with the gender and age within the oral epithelium, and thus provides a possible explanation for our previous findings on the susceptibility of elderly and female patients to SARS-CoV-2 [12]. On the one hand, this experiment was limited by the number of database samples and clinical specimens; on the other hand, it was limited by the biological test of ACE2 and TMPRSS2 expression levels from tissue specimens of patients who were not confirmed with COVID-19. And there are also many articles that have adopted similar methods to carry out some speculative experiments. An article recently published in *Nature Medicine* also predicted cell specific susceptibility to SARS-CoV-2 infection by analyzing two human oral single-cell RNA sequencing (scRNA-seq) profiles and confirmed SARS-CoV-2 infections in oral glands and mucosa. Saliva from SARS-CoV-2-infected individuals harbored epithelial cells exhibiting ACE2 and TMPRSS expression and sustained SARS-CoV-2 infection [38]. These data show that the oral cavity is an important site for SARS-CoV-2 infection, and our study can provide some supplement and explanation for the susceptibility of oral tissues to SARS-CoV-2. Li et al*.* used data collected from the cancer database to analyze the expression of ACE2 and thus speculated the susceptibility of ACE2 in diverse physiological and pathological conditions [39]. Huang et al*.* used gene expression profile-interactive analysis (GEPIA), gene set enrichment analysis (GSEA), and tumor immune estimation resource (TIMER) to compare ACE2 expression between cancer and normal tissues and the correlation between ACE2 expression and immune invasion [40].

In view of this, we used the expression trend of ACE2 and TMPRSS by analysis the data available by the database as clues, and conducted numerous biological experiments with the clinical samples we collected to explore the differences in the expression of ACE2 and TMPRSS2 in people with different ages and genders. Statistical differences in our subsequent biological experiments are significant, which partly compensate for the deficiency of statistical analysis caused by the insufficient sample size of the database. As for the subsequent biological experiments, we had also intended to divide the age more accurately, using the same groups as in Fig. 2A and B (< 50, 50–60, 60–70, and 70–80). However, the number of clinical samples collected so far is limited. When the age groups were more detailed, the sample size of each group was too small to be suitable for better statistical analysis, so only 50 years old was used as the cutoff line between the younger and the elderly. More samples and data are needed to further explore the factors affecting the expressions of ACE2 and TMPRSS2 in oral tissues, as well as the mechanisms of SARS-CoV-2 invasion and injury in oral tissues.

Another thing worth noting is about the samples we used in this study. Strictly speaking, "normal" mucosal samples should be taken from non-cancer patients. In the part of database analysis, it is difficult to find datasets for second-generation sequencing of oral epithelial tissue from a perfectly normal population in the current mainstream GEO, TCGA and SRA databases, which has brought a certain limitation to our data collection. Therefore, we use the sequencing results of the adjacent tissues to look for clues about the relationship between ACE2 and TMPRSS2 expression and age and gender. In some other studies, which have also used databases to analyze the SARS-CoV-2, susceptibility of lung, head and neck, oral cavity and other tissues, many authors were also limited to the problem of samples, using used adjacent tissues for analysis, and obtained certain results [41–43]. In order to more effectively distinguish tumor tissues and normal tissues, according to the current tumor histological classification and pathological diagnosis guidelines, the definition of peripheral tissues vary from organ to organ, which generally refers to tissues more than 2-5 cm from the edge of the cancer tissues. The goal is to ensure that the tissue is as normal as possible and can be considered non-carcinogenic. In our subsequent biological studies, we also strictly followed the histological classification specifications of tumor tissues when collecting samples. The samples collected came from patients undergoing oral cancer enlargement resection. The oral epithelial tissue ranged from below and above 2 cm from the tumor edge was collected respectively. And the tissues with a distance of more than 2 cm were used for experiments. In the concept of histomathology, the biological behavior of such adjacent tissues is different from that of cancer tissues, so as to ensure that the experimental results are as close as possible to those of normal tissues. It is undeniable that the range of canceration may be much larger than expected. Although the farther away from the cancer tissue, the closer the tissue is to normal, there may still be abnormal biological behavior of cells in the specimens we adopted, affecting the expression of ACE2 and TMPRSS2. To study the susceptibility of the two SARS-CoV-2 associated proteins, it is better to directly detect the expression level in oral mucosal of the patients diagnosed with COVID-19. In the later stage, samples from more sources and more convincing data are required, including further molecular and cytological detection.

## Conclusions

In summary, our findings have comprehensively confirmed the existence and the location of ACE2 and TMPRSS2 in oral epithelial cells from multiple levels and clarify the relationship between ACE2 and TMPRSS2 expression levels and human age and gender for the first time. The results showed that expression levels of ACE2 and TMPRSS2 were higher in the relatively elderly group than those in relatively younger group, and male oral epithelial cells exhibited higher level of TMPRSS2. Thus, the oral mucosa might be at potential risk of infection by SARS-CoV-2, especially in male or elderly patients. Using saliva to detect the nucleic acids of SARS-CoV-2 may be more accurate for elder male COVID-19 patients, who may also have relatively serious oral damage, so doctors should pay more attention to oral health care in treatment. More researches are needed to future explore the influencing factors of SARS-CoV-2 susceptibility in oral epithelial cells, as well as the mechanisms of SARS-CoV-2 invasion and injury in oral tissues.

## Supplementary Information

**Additional file 1: Table S1.** Primer sequences used in qRT-PCR.

**Additional file 2: Table S2.** General information of the clinical samples used in Figures 1, 2, 3, 4 and 5.

## Data Availability

The datasets analyzed during the current study are available from the corresponding author on reasonable request.

## Abbreviations

ACE2: Angiotensin-converting enzyme II
TMPRSS2: Transmembrane protease serine 2
qRT-PCR: Real-time quantitative polymerase chain reaction
GO: Gene ontology
