# Supplementary material for: Age and gender differences in ACE2 and TMPRSS2 expressions in oral epithelial cells
Source: J Transl Med. 2021 Aug 19;19:358. doi: 10.1186/s12967-021-03037-4 (PMC8374411; doi:10.1186/s12967-021-03037-4)
Supplement: Supplementary file 1 — Additional file 1: Table S1. Primer sequences used in qRT-PCR. [file 12967_2021_3037_MOESM1_ESM.docx]

**Table S1. Primer sequences used in qRT-PCR**

| **Primers** | **Sequence (forward/reverse)** | **Species** |
| --- | --- | --- |
| *ACE2* | 5'-GCACTCACGATTGTTGGGACT-3' 5'-TGCGGGGTCACAGTATGTTTCA-3' | Human |
| *TMPRSS2* | 5'-CTAACTGGTGTGATGGCGTGT-3' 5'-AAGTTTGGTCCGTAGAGGCGA-3' | Human |
| *GAPDH* | 5'-GGAGCGAGATCCCTCCAAAAT-3' 5'-GGCTGTTGTCATACTTCTCATGG-3' | Human |
