# Supplementary material for: Age and gender differences in ACE2 and TMPRSS2 expressions in oral epithelial cells
Source: J Transl Med. 2021 Aug 19;19:358. doi: 10.1186/s12967-021-03037-4 (PMC8374411; doi:10.1186/s12967-021-03037-4)
Supplement: Supplementary file 2 — Additional file 2: Table S2. General information of the clinical samples used in Figures 1, 2, 3, 4 and 5. [file 12967_2021_3037_MOESM2_ESM.docx]

**Table S2. General information of the clinical samples used in Figure 1-5**

| **General information of the clinical samples used in Figure 1, Figure4 and Figure 5** | | |
| --- | --- | --- |
| **Age**  >50  <50 | 32  17 | **The reason for oral surgery** |
|  |  | Oral squamous cell carcinoma |
| **Gender**  Male | 33 |  |
| Female | 16 |  |
| **General information of the clinical samples used in Figure 2 and Figure 3** | | |
| **GSE30784** | | |
| **Age**  >50  <50 | 14  31 | **The reason for oral surgery** |
|  |  | Oral tissue dysplasia/obstructive sleep apnea-hypopnea syndrome (OSAHS) |
| **Gender**  Male | 32 |  |
| Female | 13 |  |
| **GSE9844** | | |
| **Age**  >50  <50 | 8  4 | **The reason for oral surgery** |
|  |  | Tongue squamous cell carcinoma |
| **Gender**  Male | 9 |  |
| Female | 3 |  |
| **GSE42743** | | |
| **Age**  >50  <50 | 22  7 | **The reason for oral surgery** |
|  |  | Oral tumor |
| **Gender**  Male | 21 |  |
| Female | 8 |  |
